# Complete and Incomplete Resection for Progressive Glioblastoma Prolongs Post-Progression Survival

**DOI:** 10.3389/fonc.2022.755430

**Published:** 2022-02-16

**Authors:** Felix Behling, Julia Rang, Elena Dangel, Susan Noell, Mirjam Renovanz, Irina Mäurer, Jens Schittenhelm, Benjamin Bender, Frank Paulsen, Bettina Brendel, Peter Martus, Jens Gempt, Melanie Barz, Bernhard Meyer, Marcos Tatagiba, Marco Skardelly

**Affiliations:** ^1^ Department of Neurosurgery, University Hospital Tuebingen, Eberhard Karls University Tuebingen, Tuebingen, Germany; ^2^ Center for Neuro-Oncology, Comprehensive Cancer Center Tuebingen Stuttgart, University Hospital Tuebingen, Eberhard Karls University of Tuebingen, Tuebingen, Germany; ^3^ Department of Neurology and Interdisciplinary Neuro-Oncology, University Hospital Tuebingen, Eberhard Karls University of Tübingen, Tuebingen, Germany; ^4^ Institute of Pathology and Neuropathology, Division of Neuropathology, University Hospital Tuebingen, Eberhard Karls University Tuebingen, Tübingen, Germany; ^5^ Department of Neuroradiology, University Hospital Tuebingen, Eberhard Karls University Tuebingen, Tuebingen, Germany; ^6^ University Department of Radiation Oncology, University Hospital Tuebingen, Eberhard Karls University of Tuebingen, Tuebingen, Germany; ^7^ Institute of Clinical Epidemiology and Applied Biometry, University Hospital Tuebingen, Eberhard Karls University Tuebingen, Tuebingen, Germany; ^8^ Department of Neurosurgery, School of Medicine, Klinikum rechts der Isar, Technische Universität Muenchen, Muenchen, Germany; ^9^ German Cancer Consortium (DKTK), Deutsche Krebsforschungszentrum (DKFZ) Partner Site Tuebingen, Tuebingen, Germany

**Keywords:** progressive glioblastoma, surgery, re-surgery, recurrent surgery, extent of resection, post progression survival, resectability

## Abstract

**Objective:**

The role of resection in progressive glioblastoma (GBM) to prolong survival is still controversial. The aim of this study was to determine 1) the predictors of post-progression survival (PPS) in progressive GBM and 2) which subgroups of patients would benefit from recurrent resection.

**Methods:**

We have conducted a retrospective bicentric cohort study on isocitrate dehydrogenase (*IDH*) wild-type GBM treated in our hospitals between 2006 and 2015. Kaplan-Maier analyses and univariable and multivariable Cox regressions were performed to identify predictors and their influence on PPS.

**Results:**

Of 589 patients with progressive *IDH* wild-type GBM, 355 patients were included in analyses. Median PPS of all patients was 9 months (95% CI 8.0-10.0), with complete resection 12 months (95% CI 9.7-14.3, n=81), incomplete resection 11 months (95% CI 8.9-13.1, n=70) and without resection 7 months (95% CI 06-08, n=204). Multivariable Cox regression demonstrated a benefit for PPS with complete (HR 0.67, CI 0.49-0.90) and incomplete resection (HR 0.73, 95% CI 0.51-1.04) and confirmed methylation of the *O6-methylguanine-DNA-methyltransferase (MGMT)* gene promoter, lower age at diagnosis, absence of deep brain and multilocular localization, higher Karnofsky Performance Status (KPS) and recurrent therapies to be associated with longer PPS. In contrast, traditional eloquence and duration of progression-free survival had no effect on PPS. Subgroup analyses showed that all subgroups of confirmed predictors benefited from resection, except for patients in poor condition with a KPS <70.

**Conclusions:**

Out data suggest a role for complete and incomplete recurrent resection in progressive GBM patients regardless of methylation of *MGMT*, age, or adjuvant therapy but not in patients with a poor clinical condition with a KPS <70.

## Introduction

Glioblastomas (GBM) are the most common and one of the most lethal malignant brain tumors with an incidence rate of 3-4 per 100,000 inhabitants (1). Despite multidisciplinary therapy with chemoradiotherapy, the prognosis remains poor with a median overall survival (OS) of about 16 months reported in recent studies (2–4).

At the time of tumor progression, patients undergo either local therapies such as recurrent surgery and radiotherapy and/or systemic therapy depending on their age, symptom profile, general clinical condition and tumor localization. Resection is favored if the patient is in good clinical condition and the tumor is located in a well resectable area or if a significant progressive tumor mass is causing new neurological symptoms with impending escalation of intracranial pressure (5).

In GBM patients the resection of the primary tumor is associated with prolonged overall survival time (6) although the proposed resection thresholds range from maximum safe resection up to complete resection (7–11). In progressive GBM patients the role of recurrent resection is still controversial. While some studies did not demonstrate a benefit of recurrent resection at all (12, 13) others did (14–18). Age, Karnofsky Performance Status (KPS), tumor volume, extent of resection and eloquent tumor location were suggested as predictors of survival in GBM progression (19, 20). Several studies suggested complete tumor resection as an independent predictor of post-progression survival (PPS), while the role of incomplete resection is still controversial (15–18). Furthermore, currently a prospective trial is evaluating recurrent surgery in progressive glioblastoma (Schucht, clinicaltrials.gov). Nevertheless, only about 20-30 percent of patients with progressive GBM are considered for recurrent resection (21). It would be helpful in clinical routine to optimally select patients regarding probable benefit from recurrent surgery according to clinical factors.

However, as the published data remain contradictory and the resulting recommendations are still controversial, further research is warranted. Therefore, we conducted a retrospective bicentric cohort study with regard to the following topics: 1) Is there a role for resection in subgroups of progressive GBM patients; 2) do patients with progressive GBM benefit from incomplete tumor resection and 3) which subgroups of patients may benefit from resection in terms of age, KPS, *MGMT* and other predictors of survival?

## Methods

### Study Design

We conducted a retrospective bicentric cohort study on the importance of resection in patients with progressive isocitrate dehydrogenase (*IDH*) wild-type GBM. Patients from two university hospitals (Universitaetsklinikum Tuebingen, Baden-Wuerttemberg, Germany and Klinikum rechts der Isar, Technische Universität Muenchen, Germany) were included. The main clinical endpoint was post-progression, as overall survival is biased by not including patients who died before first diagnosed progression, thus estimating too long an overall survival. Survival was evaluated by Kaplan-Meier analyses and univariable and multivariable Cox regressions.

### Study Population

All patients (age ≥ 18 years) with progressive *IDH* wild-type glioblastoma who underwent surgery for the primary tumor between 2006 and 2015 in one of the participating centers were included in this study. The institutional ethics committees approved the study. We collected the following data from patients records for each patient: Age, gender, tumor site and tumor localization including eloquent brain regions, mutations of *IDH1/2* and methylation status of O6-methylguanine-DNA-methyltransferase (*MGMT*), tumor infiltration of the ventricular wall/subependymal spread, postoperative extent of resection and adjuvant treatments (radiotherapy, chemotherapy, radiochemotherapy and best supportive care) after initial and recurrent surgery, postoperative neurological deficits, Karnofsky Performance Status, time of initial diagnosis, time of progression, last visit and death. Extent of resection was determined by a neuroradiologist and a neurosurgeon using magnetic resonance imaging within 72 hours after surgery; complete resection was defined as no residual contrast enhancement. Patients with missing data of important covariates were excluded.

### Ethical Approval

All procedures were in accordance with the ethical standards of the institutional and/or national research committee and with the 1964 Helsinki declaration and its later amendments or comparable ethical standards. The present study was approved by the Ethics Committee of Tuebingen, Baden-Wuerttemberg, Germany, approval No. 115/2015BO2).

### Statistical Analyses

We analyzed the clinical endpoints using PPS and OS, which were defined as the intervals between initial diagnosis or first tumor progression (PPS) and the patient’s death (OS) or last clinical follow-up/control (censored). The patients were initially divided into three groups: A) patients with complete recurrent tumor resection; B) patients with incomplete recurrent tumor resection and C) patients without recurrent tumor resection or biopsy. Group C was subdivided further (see below).

First univariable Cox regressions evaluated established prognostic covariates (age, *MGMT* status (methylated vs. unmethylated), first and recurrent therapies (radiochemotherapy vs radiotherapy vs chemotherapy), first extent of resection (complete vs. incomplete vs no resection/biopsy), KPS, progression free survival (PFS) and potentially prognostic covariates (tumor location, traditional eloquence (yes/no), subependymal spread (yes/no), duration of progression-free-survival, use of steroids (yes/no)) in glioblastoma patients.

KPS was dichotomized by classification and regression tree (CART) analyses and adjusted to established threshold defined in the literature (≥70 vs <70). A new covariate “resectability” as recently introduced by the authors (22) based on univariable Cox regressions of tumor location. All tumors were divided into good resectable and bad resectable according to their location by the authors. Glioblastomas seated in deep areas such as the diencephalon, thalamus, basal ganglia, brain stem as well as multicentric tumors were rated as bad resectable.

**Figure 1:**
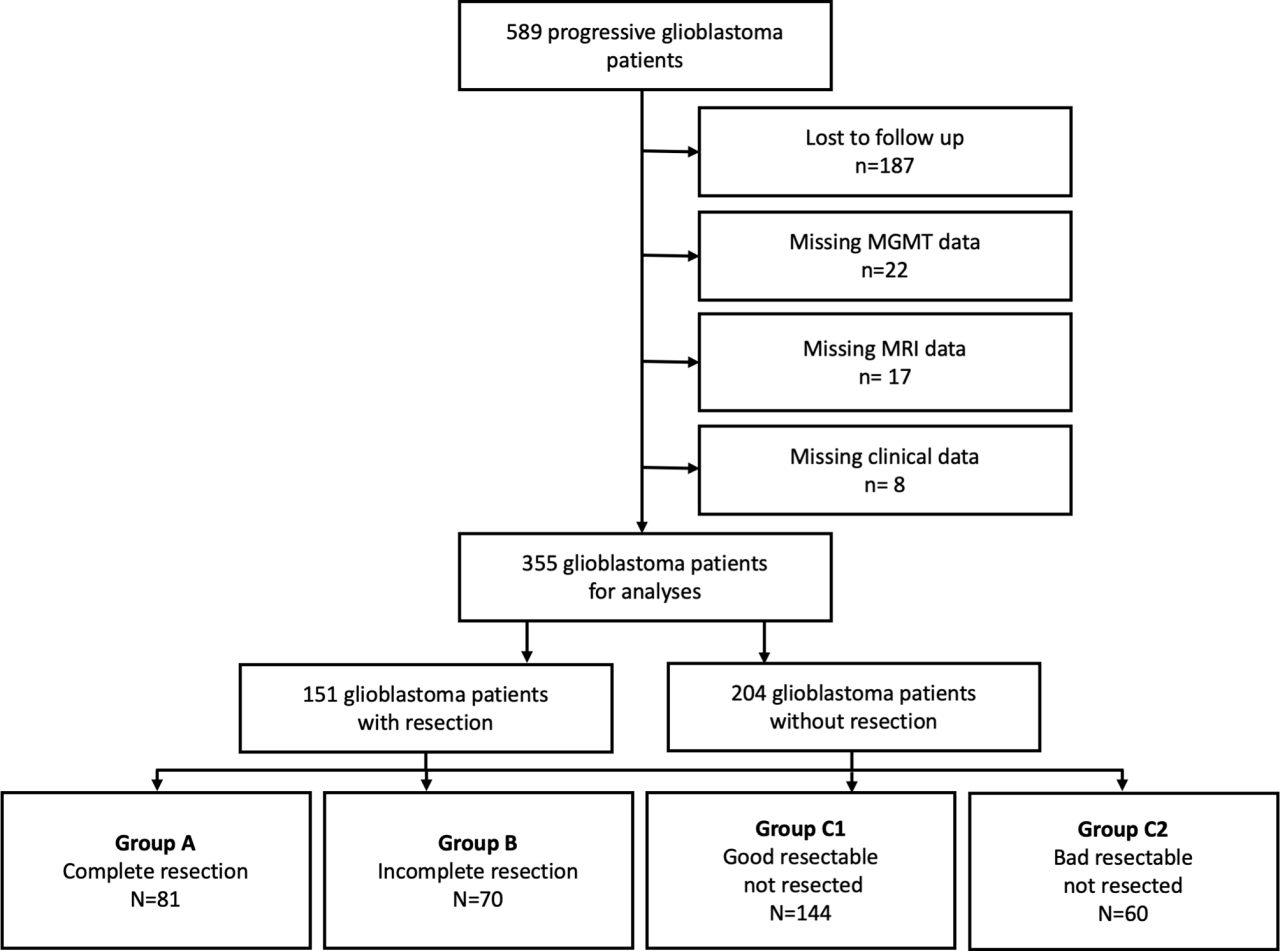
Trial profile. Flow diagram of patients with progressive. IDH-Wildtype glioblastoma.

Univariable significant covariates were included in multivariable analyses with bidirectional elimination. Interactions between covariates were evaluated and significant interactions were addressed a) by establishing a composite score for extent of resection and resectability and b) by repeating multivariable analyses stratified to KPS between KPS and recurrent therapy modality. Following the introduction of the composite score (resection/resectability), group C was divided into group C1 including patients who had not been operated on with good resectable tumors and group C2) patients with bad resectable tumors (**Figure 1**). The results were expressed as hazard ratios (HR) with 95% confidence intervals (CIs) and p-values. The goodness of fit of the model was determined by a Cox and Snell pseudo-R2 corrected for the number of covariates.

PPS and OS were analyzed by Kaplan Maier curves. Median survival times were shown in months and 95% confidence intervals (CI). We performed subgroup analyses to compare PPS between patients with recurrent resection and without recurrent resection regarding methylation of the *MGMT* promotor, age (≤60, >60), KPS-Status (≤60, 70-80, 90-100), recurrent therapy (radiotherapy, chemotherapy, radiochemotherapy and no tumor specific therapy/best supportive care) and progression free survival (<3, 3-6, 6-12, >12). These analyses were done after exclusion of patients with bad resectable (group C2) tumors to exclude the influence of tumor localization on Kaplan-Maier analyses (**Figures 3A–D** and **Table 2**).

The significance level was defined as α<0.05 (two-sided), JMP^®^ (Cary, NC: SAS Institute Inc.; 1989) Statistical Discovery Software Version 14.2 and SPSS for Windows release 26 (Armonk, NY: IBM Corp 2019) was used for statistical analysis.

## Results

### Patients

From 589 progressive *IDH* wild-type GBM patients treated in our two centers from 2006 to 2015, we had complete data sets for multivariable analyses in 355 patients. Two hundred and thirty-four patients had to be excluded due to lost follow-up (n=187), missing *MGMT* promotor status (n=22) and missing magnetic resonance imaging data (n=17) or clinical data (n=8) (**Figure 1**). At the time of analysis 303 (85%) patients had died and 52 (15%) patients were either alive or no longer available for follow-up (censored data). The clinical data of the eligible patients are presented in **Table 1** in relation to the 4 study groups A, B, C1 and C2. Eighty-one patients had a complete (group A) and 70 patients had an incomplete resection (group B). In group A, 54 patients (67%) had stable KPS, 9 patients (11%) had improved KPS, and 18 patients (22%) had worsened KPS at discharge. In group B, 41 patients (59%) had stable KPS, 9 patients (4%) had improved KPS, and 18 patients (37%) had worsened KPS at discharge. The median KPS change was 0, and the 90/10 quantiles were 0 and -20%. The remaining 204 patients did not receive a resection. In 144 of these cases the lesion was assessed as good resectable (group C1) and in 60 cases as bad resectable (group C2). At disease progression, 204 patients received chemotherapy, 55 patients received radiotherapy, 28 patients received radio- and chemotherapy, and 68 patients received no therapy. After disease progression, most patients (103) received retreatment with temozolomide, either standard (5/28: 63 patients) or intensified protocol (7/7: 40 patients). Thirty-one patients received lomustine, 8 patients nimustine, and 14 patients other drugs or data not available in 47 patients.. Radiation therapy for progressive disease was highly variable and depended, among other factors, on the primary treatment, i.e., patients who had received first-line chemotherapy because of their age and methylation of MGMT received 34 Gy of radiotherapy in 10 fractions, and some received 60 Gy in 30 fractions. Other patients who had also received first-line radiotherapy received radiotherapy at a dose of 20 Gy, for example, in 5 x 4 Gy or 4 x 5 Gy fractions as the disease progressed.

**Table 1:** Patients characteristics.

	Resection/Resectability				
	A	B	C1	C2	All
**Number of patients**	81 (22%)	70 (18%)	144 (43%)	60 (17%)	355 (100%)
**Gender**					
female	27 (33%)	24 (34%)	60 (42%)	20 (33%)	131 (37%)
male	54 (67%)	46 (66%)	84 (58%)	40 (67%)	224 (63%)
**Age**					
Median	56	55	65	59	59
Quantile_25	51	49	55	54	51
Quantile_75	69	63	64	69	75
Mean	58	55	63	58	59
SD	12	12	12	11	12
≤50	18 (22%)	25 (36%)	22 (15%)	11 (18%)	76 (21%)
>50	63 (78%)	45 (64%)	122 (85%)	49 (82%)	279 (79%)
≤65	55 (68%)	57 (81%)	77 (53%)	47 (78%)	236 (66%)
>65	26 (32%)	13 (19%)	67 (47%)	13 (22%)	119 (34%)
**First Resection**					
Complete	49 (60%)	34 (49%)	73 (51%)	28 (47%)	184 (52%)
Incomplete	30 (37%)	35 (50%)	56 (39%)	20 (33%)	141(40%)
Biopsy	2 (2%)	1 (1%)	14 (10%)	12 (20%)	29 (8%)
**First therapy modality**					
Radiotherapy	11 (14%)	8 (11%)	46 (32%)	11 (18%)	76 (21%)
Chemotherapy	0	3 (4%)	19 (13%)	4 (7%)	26 (7%)
Radiochemotherapy	61 (75%)	49 (70%)	62 (43%)	40 (67%)	212 (60%)
No therapy, study arm	9 (11%)	10 (14%)	17 (12%)	5 (8%)	41 (12%)
**MGMT**					
methylated	25 (31%)	27 (39%)	59 (41%)	20 (33%)	131 (37%)
unmethylated	56 (69%)	43 (61%)	85 (59%)	40 (67%)	224 (63%)
**Side**					
right	44 (54%)	36 (51%)	74 (51%)	31 (52%)	185 (52%)
left	37 (46%)	34 (49%)	67 (47%)	22 (37%)	160 (45%)
bilateral	0	0	3 (2%)	7 (12%)	10 (3%)
**Tumor localization at progression**					
frontal	19 (24%)	14 (20%)	33 (23%)	0	66 (19%)
precentral	2 (2,5%)	3 (4%)	8 (5,5%)	0	13 (4%)
postcentral	2 (2,5%)	2 (3%)	1 (1%)	0	5 (1%)
central (precentral & postcentral)	4 (5%)	8 (12%)	11 (7,5%)	0	23 (7%)
fronto-temporal	0	0	4 (3%)	0	4 (1%)
parietal	5 (6%)	8 (12%)	12 (8%)	0	25 (7%)
parieto-temporal	6 (7,5%)	3 (4%)	4 (3%)	0	13 (4%)
parieto-okzipital	6 (7,5%)	4 (6%)	1 (1%)	0	11 (3%)
temporo-fronto-insular	4 (5%)	5 (7%)	6 (4%)	0	15 (4%)
temporo-occipital	3 (4%)	2 (3%)	6 (4%)	0	11 (3%)
occipital	1 (1%)	3 (4%)	7 (5%)	0	11 (3%)
diencephalic	1 (1%)	0	0	5 (8%)	6 (1%)
> 2 lobes	1 (1%)	0	11 (7,5%)	0	12 (3%)
multicentric	0	2 (3%)	0%	49 (82%)	51 (15%)
temporal	27 (33%)	15 (21%)	39 (27%)	0	81 (22%)
insular	0	1 (1%)	1 (0,5%)	0	2 (1%)
basal ganglia	0	0	0	3 (5%)	3 (1%)
thalamic	0	0	0	3 (5%)	3 (1%)
**Tumor infiltrating ventricular wall**					
**At progression**					
No	50 (63%)	44 (63%)	84 (59%)	15 (25%)	193 (55%)
Yes	31 (38%)	26 (37%)	59 (41%)	45 (75%)	160 (45%)
**Resectability at progression**					
Bad	1 (1%)	2 (3%)	0	60 (100%)	63 (18%)
Good	80 (99%)	68(97%)	144 (100%)	0	290 (82%)
**Time to first progression**					
Median in months	8	6	6	6	7
95% CI in months	6-9	4-8	4-8	6-7	6-7
≤ 3months	14 (17%)	24 (34%)	28 (20%)	12 (20%)	78 (23%)
> 3months - ≤ 6months	21 (26%)	13 (19%)	49 (34%)	12 (20%)	95 (25%)
> 6months - ≤ 12mnths	25 (31%)	18 (26%)	48 (33%)	24 (40%)	115 (32%)
> 12months	21 (26%)	15 (21%)	19 (13%)	12 (20%)	67 (20%)
**Eloquence at progression**					
not eloquent	42 (53%)	27 (39%)	82 (57%)	17 (28%)	168 (47%)
central (motoric/sensoric)	15 (18%)	11 (15%)	22 (15%)	6 (10%)	54 (15%)
Broca’s speech area	2 (2%)	9 (13%)	8 (6%)	3 (5%)	22 (7%)
Wernicke’s speech area	13 (16%)	12 (17%)	17 (12%)	1 (2%)	43 (12%)
Inferior parietal lobule	4 (5%)	0	6 (4%)	1 (2%)	11 (3%)
primary visual cortex	5 (6%)	9 (13%)	4 (3%)	1 (2%)	19 (5%)
Deep brain	0	2 (3%)	5 (3%)	31 (51%)	38 (11%)
**Karnofsky performance status at progression**					
Median	90	80	80	70	80
Quantile_25	80	70	62,5	50	70
Quantile_75	90	90	90	90	90
>= 70	76 (94%)	60 (86%)	108 (75%)	35 (58%)	279 (79%)
<70	5 (6%)	10 (14%)	36 (25%)	25 (42%)	76 (21%)
**Therapy modality at progression**					
Radiotherapy	8 (10%)	11 (16%)	27 (19%)	9 (15%)	55 (15,5%)
Chemotherapy	52 (64%)	26 (37%)	85 (59%)	41 (68%)	204 (57,5%)
Radiochemotherapy	7 (9%)	14 (20%)	6 (4%)	1 (2%)	28 (8%)
No therapy, best supportive care	14 (17%)	19 (27%)	26 (18%)	9 (15%)	68 (19%)

### Post Progression Survival, Overall Survival and Resectability

**Figure 2:**
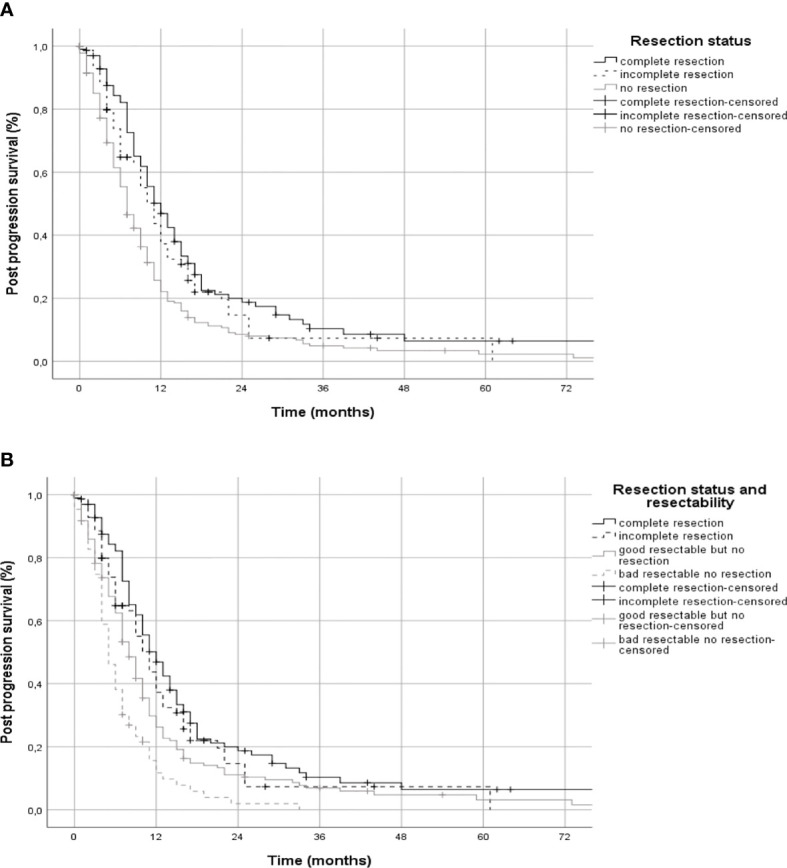
Post-progression survival by extent of resection. Panel **(A)** shows post-progression survival in Kaplan-Meier estimates for patients stratified by complete, incomplete, and no resection. In panel **(B)** the group of patients who had no resection was divided into those with either good resectable or bad resectable tumors.

Median PPS of all patients was 9 months (95% CI 8.0-10). For patients who received a recurrent resection PPS was 11 months (95% CI 10-13, group A&B)), with 12 months (95% CI 9.7-14.3) for complete recurrent resection (group A) and 11 months (95% CI 8.9-13.1) for incomplete recurrent resection (group B). Patients without a recurrent resection (group C) had a median PPS of 7 months (95% CI 06-08) (**Figure 2A**). Sixty of 63 patients considered bad resectable according to the introduced resectability score were not operated on, 2 patients received an incomplete and 1 patient a complete resection. Median PPS was 8 months (95% CI 6.9-9.1) in group C1 and 5 months (95% CI 3.8-6.2) in group C2 (**Figure 2B**).

**Figure 3:**
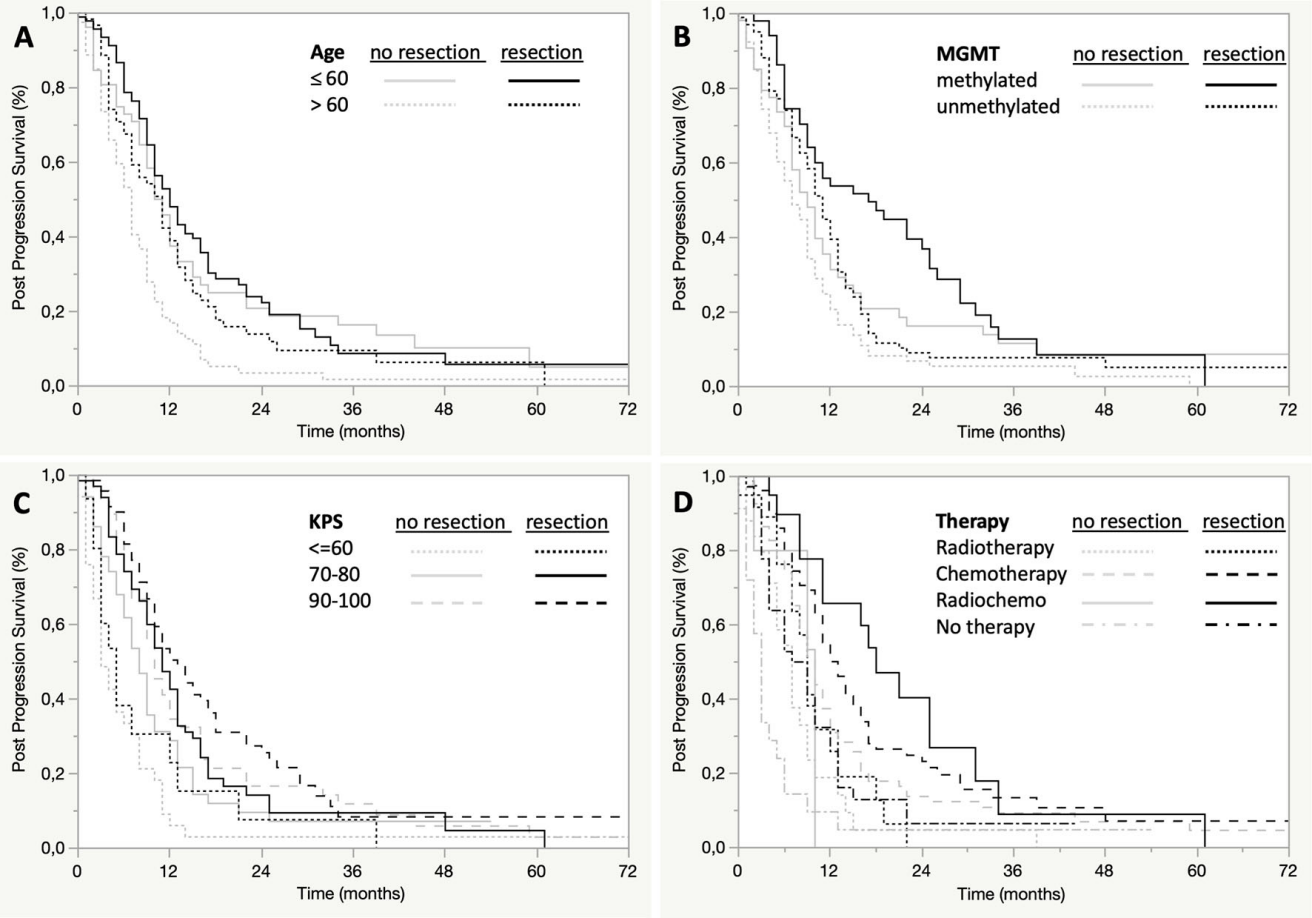
Subgroup analysis of post-progression survival by age, MGMT, KPS, and adjuvant therapy. Post-progression survival is presented in Kaplan-Meier estimates, each bivariately stratified by extent of resection and age **(A)**, MGMT methylation **(B)**, KPS **(C)**, or adjuvant therapy **(D)**.

Subgroup analyses showed that patients benefited from resection independent of their methylation of *MGMT* promotor, age, duration of progression free survival and recurrence therapy (**Figures 3A, B, D** and **Table 2**). In contrast, only patients with a KPS of ≥70 benefited from re-resection. (**Figure 3C** and **Table 2**). In addition, patients older than 60 years who underwent recurrent resection showed a greater benefit in PPS than younger patients (median difference in PPS of 4 months vs 1 month) (**Figure 3A** and **Table 2**).

**Table 2:** Post-progression survival as a function of recurrent resection performed and covariates.

Resection	No	Yes
	median months (95% CI)	
**MGMT**		
methylated	9 (7-11)	17 (9-25)
unmethylated	7 (5-9)	11 (9-12)
**Age**		
≤ 60	11 (8-13)	12 (10-15)
> 60	7 (5-8)	11-(7-13)
**KPS**		
≤ 60	4 (3-7)	5 (2-12)
70-80	8 (6-9)	11 (9-13)
90-100	10 (9-12)	13 (10-17)
**Therapy**		
Radiotherapy	7 (4-9)	9 (5-13)
Chemotherapy	10 (8-11)	13 (11-15)
Radiochemotherapy	10 (2-10)	18 (11-25)
No therapy, best supportive care	3 (1-4)	8 (4-10)

MGMT, O6-methylguanine-DNA-methyltransferase; KPS, Karnofsky Performance Status.

Median overall survival of all progressive patients was 17 months (95% CI 15-18), for patients with complete recurrent resection (group A) 22 months (95% CI 19-29), with incomplete recurrent resection (group B) 18 months (95% CI 16-21) and for patients without recurrent resection for group C1 15 months (95% CI 14-17) and group C2 13 months (95% CI 11-16), respectively. Patients without progression within the observation period were not included in this study.

Some relevant clinical parameters were not well balanced between the study groups. Group C2 showed significant differences to the other groups regarding tumor localization, eloquence, subependymal spread, resectability, KPS due to the intrinsic concept of the variable “resectability”. In addition, the age and frequency of initial and recurrent treatments were also not balanced between the groups, e.g. 32% (group A), 19% (group B), 47% (group C1), and 22% (group C2) of the patients were older than 65years (**Table 1**).

### Factors Associated With Benefit From Resection, Univariable Analysis

Univariable Cox regressions suggested following covariates as significant predictors of PPS: recurrent resection (HR 0.61, 95% CI 0.48-0.76, p<0.0001); a) complete resection (risk ratio (HR) 0.55 (95% CI 0.43-0.71, p<0.0001) and b) incomplete resection (HR 0.68, 95% CI 0.51-0.91, p<0.008) compared to no resection/biopsy; resectability (HR 0.53, 95% CI 0.41-0.70, p<0.0001); methylated *MGMT* promotor (HR 0.67, 95% CI 0.54-0.83, p=0.0002); age at diagnosis (per decade decrease in HR 0.83, 95% CI 0.75-0.92, p=0.0002; KPS ≥70 (HR 0.42, 95% CI 0.33-0.54, p<0.0001), PFS (per month HR 0.97, 95% CI 0.96-0.98, p<0.0001); absence of subependymal spread (HR 0.70, 95% CI 0.57-0.87, p=0012); recurrent a) radiochemotherapy (HR 0.33 (95% CI 0.36-0.77, p<0.0001), b) chemotherapy (HR 0.48 (95% CI 0.37-0.62, p<0.0001) and c) radiotherapy (HR 0.73 (95% CI 0.51-1.04, p=0.08) compared to best supportive care in progressive disease. Resectability and extent of resection showed significant interaction (p=0.02) as well as KPS and recurrence therapy modality (p=0.003).

### Multivariable Analysis Regarding the Prognostic Factors

The final multivariable Cox regression model (R2 = 0.28, Chi2 = 117.696, n=355) showed a clear risk reduction for PPS after both complete resection (group A, HR 0.67, 95% CI 0.49-0.90, p=0.009) and incomplete resection (group B; HR 0.73, 95% CI 0.51-1.04, p=0.08) compared to no resection of good resectable tumors (group C1). Patients with bad resectable tumors showed an increased risk for death (group C2, HR 1.84, 95% CI 1.31-2.17, p<0.0001) compared to patients with good resectable tumors who had no recurrent surgery (**Table 3**).

**Table 3:** Cox regression model.

	HR	95% CI		df	p
		Lower	Upper		
Reference: no resection but good resectable				3	<0.001
Complete resection	0.667	0.494	0.902	1	0.009
Incomplete resection	0.731	0.513	1.041	1	0.082
No resection, bad resectable	1.839	1.310	2.581	1	<0.001
MGMT-unmethylated	Reference				
MGMT-methylated	0.593	0.765	0.460	1	<0.001
Age at diagnosis (decrease per decade)	0.853	0.766	0.949	1	0.003
KPS at progression <70	Reference				
KPS at progression ≥70	0.234	0.135	0.404	1	<0.001
Reference: no therapy				3	<0.001
Radiotherapy	0.264	0.121	0.575	1	0.001
Chemotherapy	0.192	0.109	0.337	1	<0.001
Radiochemotherapy	0.109	0.014	0.832	1	0.03
					
					
KPS70 at progression *				3	0.003
Therapy arm					
KPS70 at progression *	3.954	1.622	9.637	1	0.002
Radiotherapy					
KPS70 at progression *	3.035	1.577	5.841	1	0.001
Chemotherapy					
KPS70 at progression *	5.500	0.671	45.056	1	0.112
Radiochemotherapy					

MGMT, O6-methylguanine-DNA-methyltransferase; KPS, Karnofsky Performance Status. KPS70 at progression * therapy arm show the interactions between the patient’s condition and the therapies used.

Furthermore, multivariable Cox regression confirmed the prognostic role of methylation of the *MGMT* gene promoter (HR 0.59, p>0.0001), age at diagnosis (per decade decrease, decrease in HR 0.85, p=0.003), KPS ≥70 at progression (HR 0.23, p <0.0001) and recurrent a) radiochemotherapy (HR 0.11, p=0.03), b) chemotherapy (HR 0.19, p<0.0001) and c) radiotherapy (HR 0.26, p=0.001) at progression were associated with PPS (**Table 3**). Progression-free survival, subependymal spread and the traditional eloquent regions had no independent effect on PPS.

**Figure 4:**
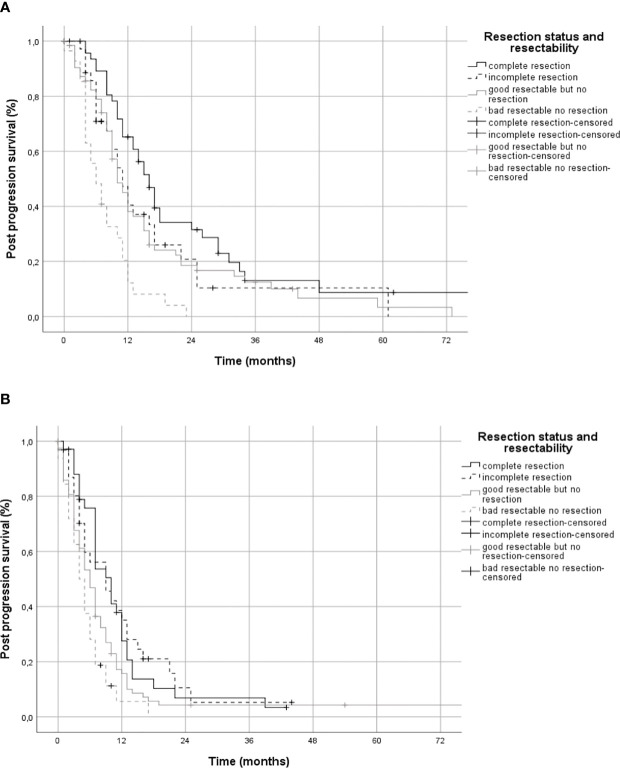
Post-progression survival in patients with favorable and unfavorable constellation of covariates. Panel **(A)** shows post-progression survival in Kaplan-Meier estimates for patients with a favorable and panel **(B)** for an unfavorable constellation of the covariates age, MGMT, KPS, and adjuvant therapy stratified by extent of resection.

Post-progression survival, analyzed using Kaplan-Meier curves separately for patients with a favorable or unfavorable constellation of covariates (age, therapy, KPS and *MGMT*), showed different effects for patients with incomplete resection (**Figures 4A, B**). Patients with a favorable prognosis showed almost the same PPS with incomplete resection (11 months, CI 95% 8-14) as patients with non-resected good resectable tumors (10 months, CI 95% 8-12, **Figure 4A**). In contrast, patients with a poor prognosis with incomplete resection (9 months, CI 95% 4-14) showed better PPS than patients with non-resected good resectable tumors (6 months, CI 95% 5-7, **Figure 4B**).

## Discussion

The role of resection in progressive GBM patients remains controversial. The question arises, which subgroups of patients might benefit from recurrent resection in terms of extent of resection, age, KPS, PFS, recurrent therapies and molecular markers. We performed Kaplan-Meier and Cox regression analyses on a retrospective bicentric cohort of 355 progressive *IDH* wild-type GBM patients and observed that patients in a good clinical condition (KPS) may benefit from complete and incomplete recurrent resection.

### Patients Population

The median time to progression in our cohort was 7.0 months, which was very similar to the original study by Stupp et al. of 6.9 months (23) and the 7.1 months observed in study of 516 GBM patients by Helseth et al. (24) Median PPS was 9 months in our study, which is also in line with the 8.5 months observed by Helseth et al. (24).

### Association of Recurrent Resection and PPS

In our study, patients who received recurrent surgery after tumor progression had a survival benefit of 4 months compared to patients who received no further surgery, which was also observed by Wann et al (14). We observed clear differences in PPS times between the different patient groups (A-C2). Patients with complete and incomplete resection showed a significant survival advantage with a risk reduction of approximately 30% with hazard ratios of 0.67 and 0.73, respectively, in multivariable Cox regression compared to good resectable patients who did not receive recurrent surgery. Although statistical analysis narrowly missed significance for incomplete resection (HR 0.73, 95% CI 0.51-1.04, p=0.08, n.s.), the comparable HR of complete resection of 0.67 most likely suggests that it was missed due to an insufficient number of cases. This is in partial contrast to the retrospective observation of the prospective “DIRECTOR” study, which only showed a survival benefit for complete but not for incomplete tumor resection (15). Other studies also showed that not only complete tumor resection, but also a small residual tumor volume (<3cm^3^) (16) or NTR (near total resection, i.e. only marginal enhancement of the resection cavity) (18) were associated with improved survival.

Based on our multivariable regression we investigated the effects of resection extent on PPS in patients with a favorable and a unfavorable covariate constellation (**Figure 4**). We found that patients with a favorable covariate constellation did not benefit from incomplete resection (group B, median PPS 11 months) compared to patients with good resectable tumors that were not resected (group C1, median PPS 10 months, **Figure 4A**), but patients with a unfavorable covariate constellation benefited from incomplete resection (group B, median PPS 9 months) compared to patients good resectable but not resected (group C1, median PPS 6 months, **Figure 4B**).

### Association of Progressive Tumor Location With PPS and KPS

Traditional eloquent tumor localization has been proposed as an independent predictor of survival in progressive GBM (17, 20). We could not confirm this observation when we applied electrophysiology and awake surgery in traditionally eloquent regions as we routinely do, but instead we identified a deep brain or multilocular location of the progressive tumor as an independent risk factor for shorter survival in our patient cohort, which we recently introduced as a new covariate that we called “resectability” (22). Despite routine electrophysiology and awake surgery to prevent new postoperative deficits, we observed new deficits at discharge in nearly 1/3 (29%) of all patients, but in most cases they were mild (-10% to -20%). Nevertheless, the impact on quality of life from the risk of new deficits must also be considered when evaluating the chance of extending survival with recurrent resection.

### Association of Age, KPS, Recurrent Therapy and *MGMT* Methylation With Post-Progression Survival

We confirmed age as a prognostic variable for PPS in patients with progressive GBM, but not as a predictive variable for benefit from recurrent surgery. With increasing age, PPS decreased, but also older patients showed a benefit of recurrent resection, as observed by Stark et al. (25). Furthermore, we observed that the median difference in PPS was even higher in re-resected older patients (>60 years, 4 months) compared with patients who were not resected than in younger patients (<60 years, 1 months) (**Figure 3A** and **Table 2**). This is probably due to a selection bias, since in progressive GBM 50% of younger patients (≤60 years) were resected and only 35% of older patients (>60 years). All subgroups of GBM patients stratified by *MGMT*, extent of first resection, therapies after progression and good KPS also benefited from recurrent resection with the exception of patients in poor clinical condition with a KPS <70. Therefore, only prognostic but not predictive roles for recurrent resection can be derived for these covariates.

### Impact of Progression Free Survival on Post-Progression Survival

Dirks et al. reported 1993 that patients with GBM who had a recurrence later than 50 weeks after the first resection showed a significantly longer OS. PFS was therefore proposed as an independent variable for survival (26). We could not confirm duration of PFS as an independent predictor of PPS when the molecular markers *IDH* and *MGMT* were considered. Although GBM patients with PFS > 12 months also had longer PPS, this could be attributed to a more likely methylated *MGMT* promotor, e.g. the patient groups with ≤3 months and >12 months PFS had a methylation rate of *MGMT* of 28% and 46%. Since the established molecular biomarkers were not known by the time of the study by Dirks et al., it is more likely that the two groups stratified by PFS of 50 weeks had different rates of *IDH*-mutated and *MGMT*-methylated tumors.

### Limitations and Strengths of the Study

The retrospective design is one of the main limitations of this study. Also, the determination of molecular markers such as mutation in the *IDH* gene or methylation of the *MGMT* promotor was not performed centrally according to a defined protocol, but locally in the two participating centers. Since tumor volumes were not determined by volumetry, but patients were stratified into groups with complete and incomplete resection or no resection at all, we had to limit the analyses of survival to semi-parametric methods and were not able to identify a possible threshold of residual tumor volume that would be associated with prolonged survival. Due to the retrospective design, the involved predictors of PPS are neither randomized nor stratified and are therefore not balanced in the investigated groups limiting the generalizability of the data. Nevertheless, our cohort of 355 patients with progressive *IDH* wild-type GBM reflects the heterogeneity of the GBM patients in clinical routine and in our opinion the data represent this population in a realistic manner. Furthermore, the well-defined clinical and molecular data sets accounts for the uneven distribution of covariates to a large extent by performing multivariable regression including the covariates in addition to the univariable survival analyses.

## Conclusions

1) Recurrent resection plays a distinct role in the therapy of selected progressive GBM patients
2) Our data suggest that both complete and incomplete resection may contribute to prolongation of PPS in selected progressive GBM patients.
3) Recurrent resection should be considered in patients with progressive GBM, regardless of age, methylation of *MGMT* or planned recurrent therapy presenting with KPS ≥70 at the time of progression
4) In our patient cohort progression free survival and traditional eloquence were not associated with survival but tumors in a deep brain or multilocular location.

## Data Availability Statement

The raw data supporting the conclusions of this article will be made available by the authors, without undue reservation.

## Ethics Statement

The studies involving human participants were reviewed and approved by Ethics Committee of Tuebingen, Baden-Wuerttemberg, Germany. Written informed consent for participation was not required for this study in accordance with the national legislation and the institutional requirements.

## Author Contributions

Conception and experimental design: FB and MS. Data acquisition: JR, ED, SN, IM, JS, BBe, FP, MR, JG, and MB. Analysis: MS, FB, BBr, and PM. Interpretation of the data: MS, FB, IM, JS, BBe, FP, BBr, PM, JR, MR, BM, and MT. Drafting: MS, FB, and PM. Revision: JR, ED, JS, IM, BBe, FP, MR, JG, MB, JS, BM, and MT. All authors contributed to the article and approved the submitted version.

## Conflict of Interest

The authors declare that the research was conducted in the absence of any commercial or financial relationships that could be construed as a potential conflict of interest.

## Publisher’s Note

All claims expressed in this article are solely those of the authors and do not necessarily represent those of their affiliated organizations, or those of the publisher, the editors and the reviewers. Any product that may be evaluated in this article, or claim that may be made by its manufacturer, is not guaranteed or endorsed by the publisher.

## Abbreviations

GBM, Glioblastoma; IDH, Isocitrate dehydrogenase; KPS, Karnofsky performance status; MGMT, O6-methylguanine-DNA-methyltransferase; PFS, Progression free survival; PPS, Post-progression survival.
